# Supplementary material for: En route to dynamic life processes by SNARE-mediated fusion of polymer and hybrid membranes
Source: Nat Commun. 2021 Aug 17;12:4972. doi: 10.1038/s41467-021-25294-z (PMC8371082; doi:10.1038/s41467-021-25294-z)
Supplement: Supplementary file 1 — Supplementary Information [file 41467_2021_25294_MOESM1_ESM.pdf]

Supplementary Information for

En route to dynamic life processes by SNARE-mediated fusion of polymer and hybrid membranes

Lado Otrin, Agata Witkowska, Nika Marušič, Ziliang Zhao, Rafael B. Lira, Fotis L. Kyrilis, Farzad Hamdi, Ivan Ivanov, Reinhard Lipowsky, Panagiotis L. Kastritis, Rumiana Dimova, Kai Sundmacher, Reinhard Jahn, Tanja Vidaković-Koch

Correspondence to: otrin@mpi.magdeburg.mpg.de

This file includes:

Figs. S1 to S15

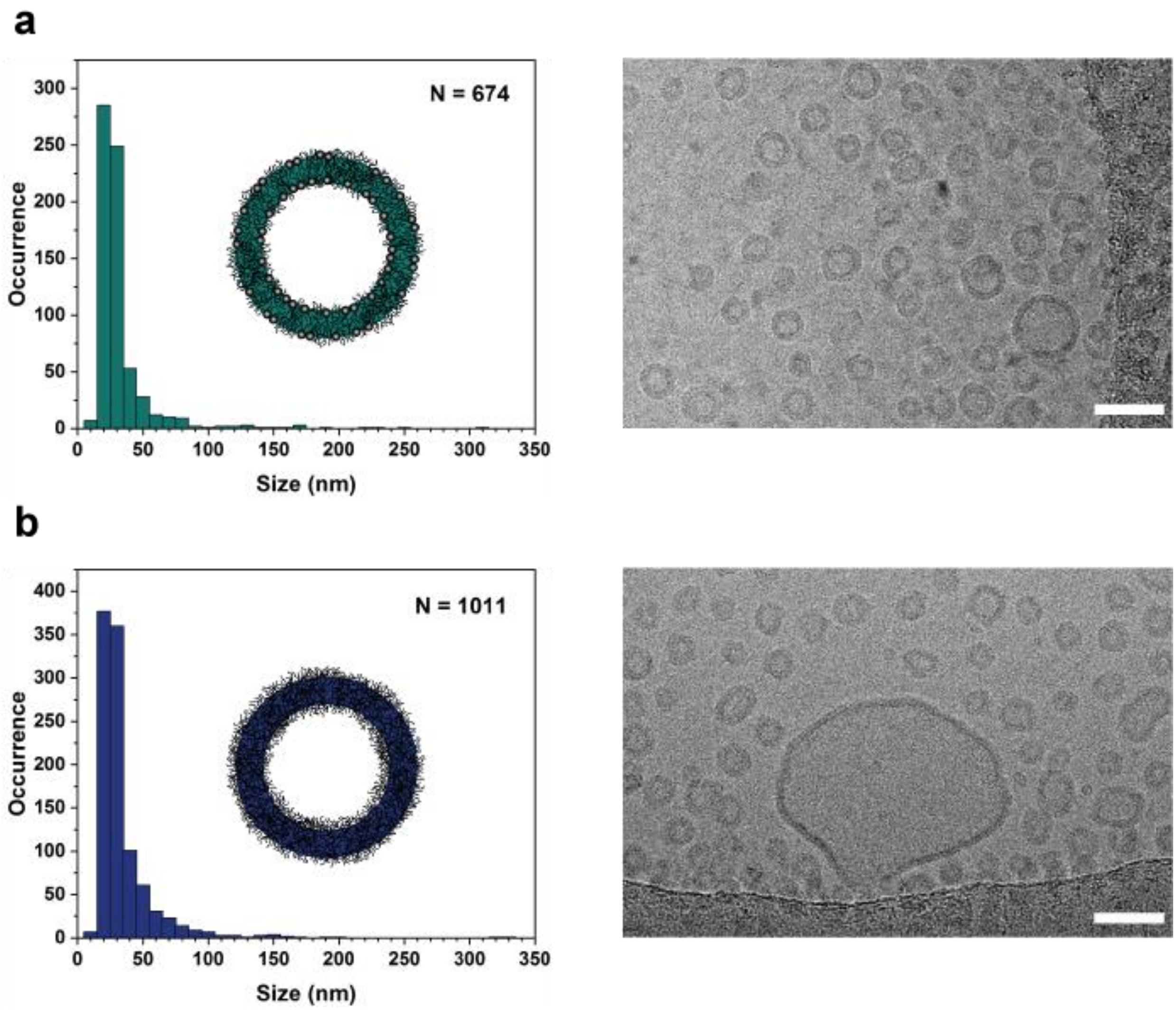

Fig. S1. Size distribution (left) of SNAREs-containing hybrid (a) or polymer (b) vesicles as determined from cryo-EM micrographs (right). Indicated is the number of counted vesicles (*N*). Scale bar = 50 nm.

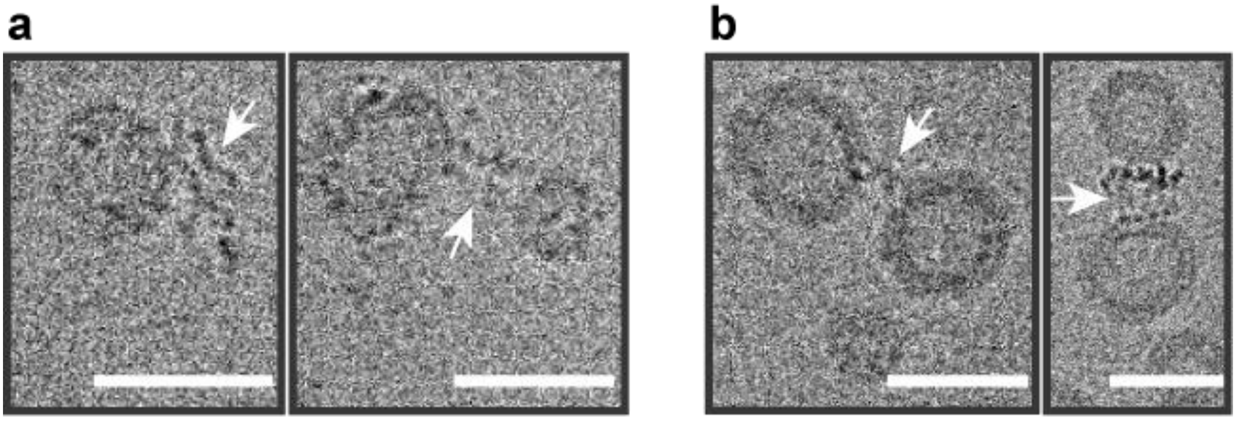

Fig. S2. Reconstitution of SNAREs into polymersomes and hybrids. SNAREs, indicated with white arrows, were inserted into polymer (a) and hybrid (b) vesicles via detergent removal by size exclusion chromatography. Scale bars = 30 nm.

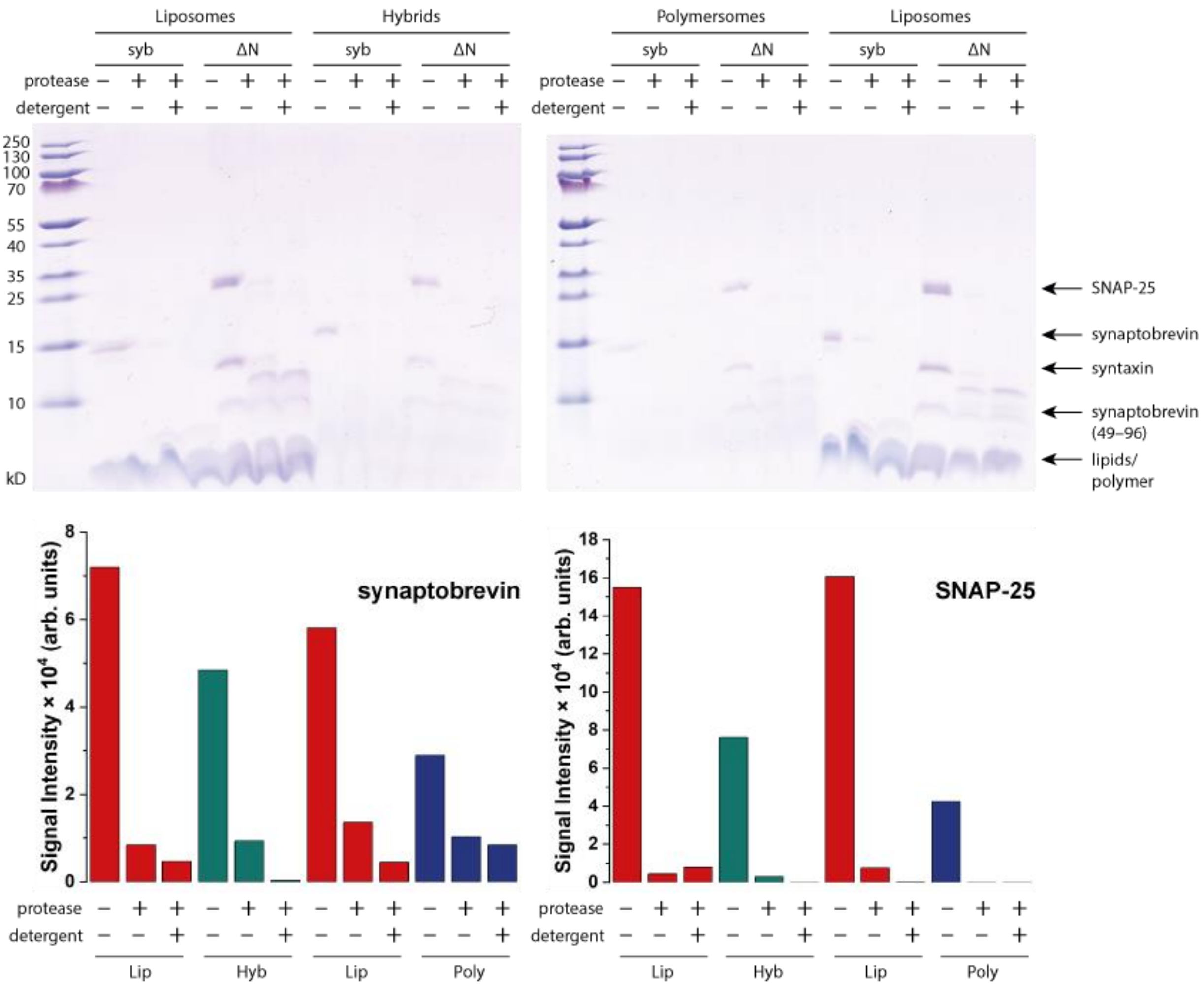

Fig. S3. Orientation of SNAREs in different types of proteovesicles. Lipid (Lip), polymer (Poly) and hybrid (Hyb) vesicles reconstituted with either synaptobrevin (syb) of ΔN complex (ΔN) were treated with protease (trypsin) in the absence (digested are outwards-facing SNAREs only) or presence (digested are inwards- as well as outwards-facing SNAREs) of detergent, which was used to dissolve the vesicles. SNAREs content in treated samples along with the total initial amount of SNAREs (untreated samples) were analysed by SDS-PAGE and compared to determine the fraction of outwards-facing SNAREs. Examples of the measured band intensities of treated and untreated samples are shown in the lower two panels.

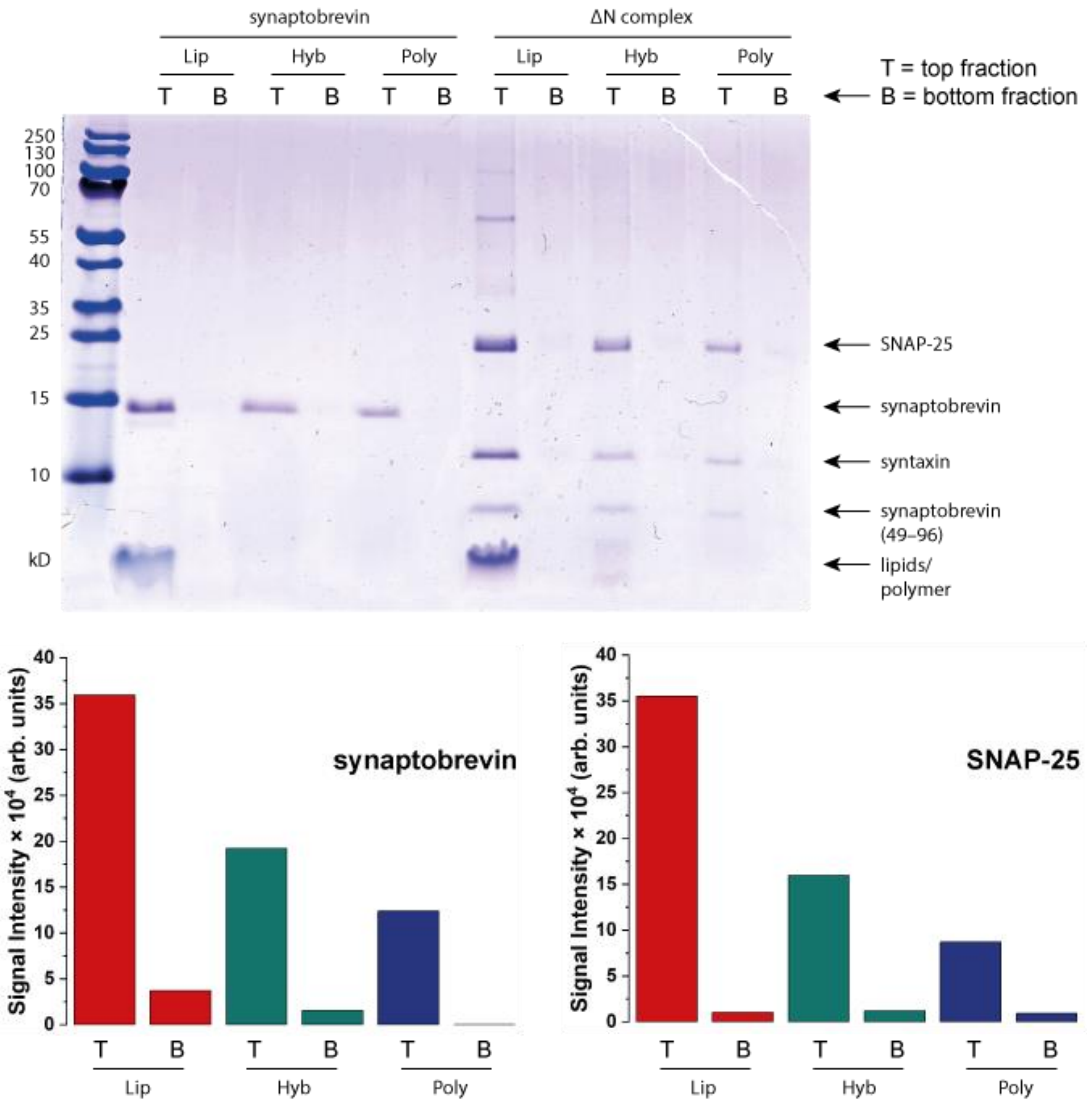

Fig. S4. Assessment of integration stability and integration efficiency of SNAREs in different proteovesicles. Lipid (Lip), polymer (Poly) and hybrid (Hyb) vesicles reconstituted with either synaptobrevin (syb) of ΔN complex (ΔN) were analysed on a discontinuous Nycodenz gradient, whereby following ultracentrifugation, SNAREs-containing vesicles partitioned in the uppermost (buffer) layer of the gradient, while non-incorporated SNAREs as well as disassembled ΔN complex could be found in bottom layer. Protein content of top and bottom layers were analyzed via SDS-PAGE and the related band intensities (shown in the lower two panels) were compared to determine the fraction of firmly inserted SNAREs.

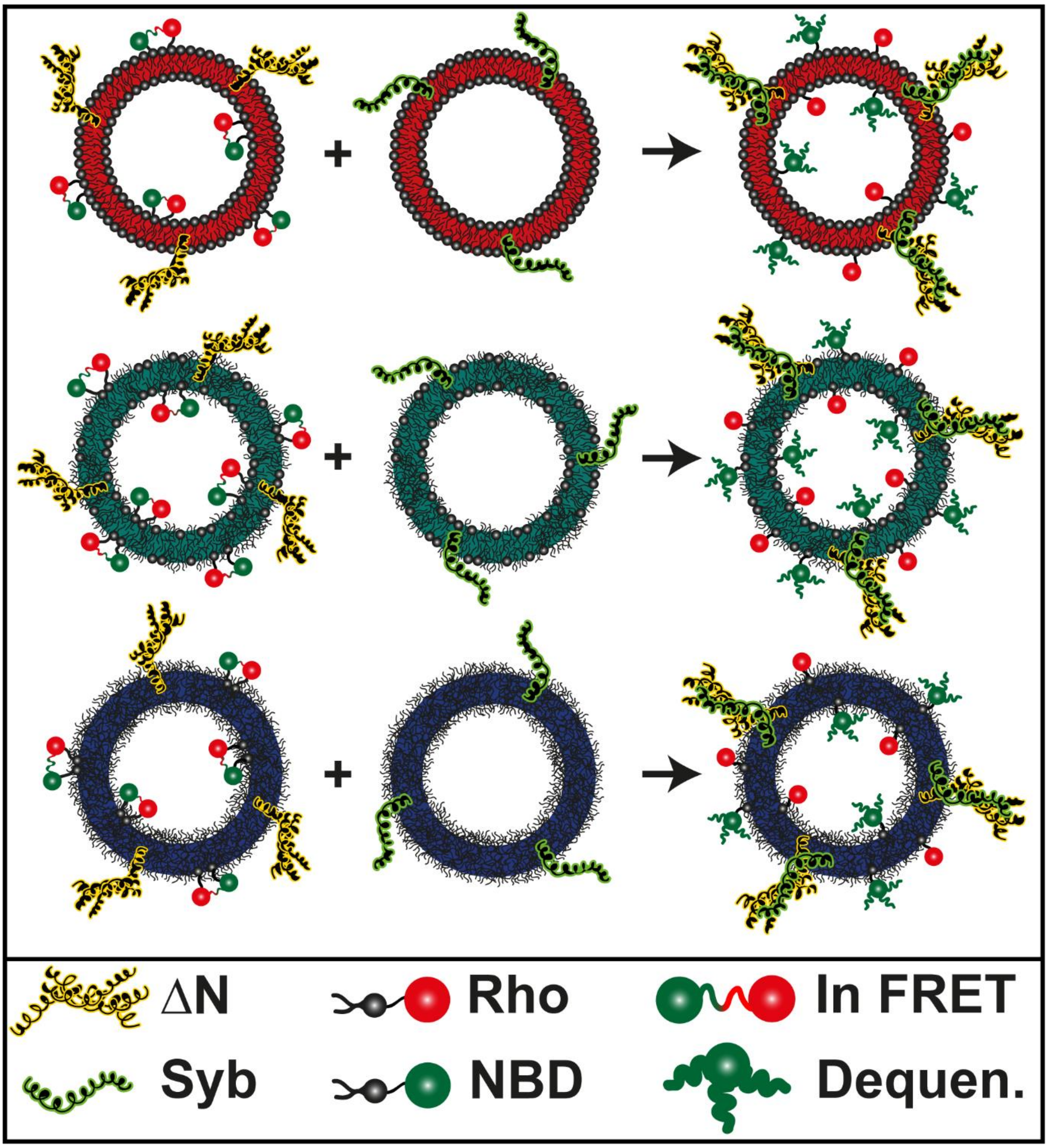

Fig. S5. Schematic representation of the SNARE-mediated membrane mixing setup. For this purpose, two separate populations of lipid (red), hybrid (green) or polymer (blue) proteovesicles were formed. One population contained the FRET couple Rho/NBD and was reconstituted with ΔN complex. In this population, the emission of NBD was quenched via proximal Rho. The second population contained no dyes and was reconstituted with synaptobrevin. Following fusion, dye-containing membranes were diluted with the dye-free membranes, resulting in NBD dequenching and increase in fluorescence emission.

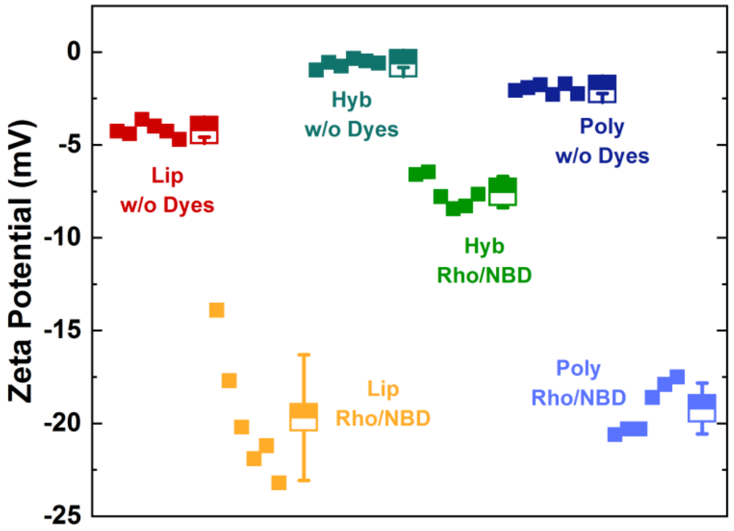

Fig. S6. Zeta potential of lipid (Lip), polymer (Poly) and hybrid (Hyb) vesicles, intended for membrane mixing in milli-Q water. Dye free as well as dye-containing (Rho/NBD) vesicles were prepared in milli-Q water and the zeta potential was measured at 37 °C. Separate measurements with mean values and standard deviations are shown. The added lipid dyes contributed significantly to the overall membrane charge, but in particular in liposomes and polymersomes.

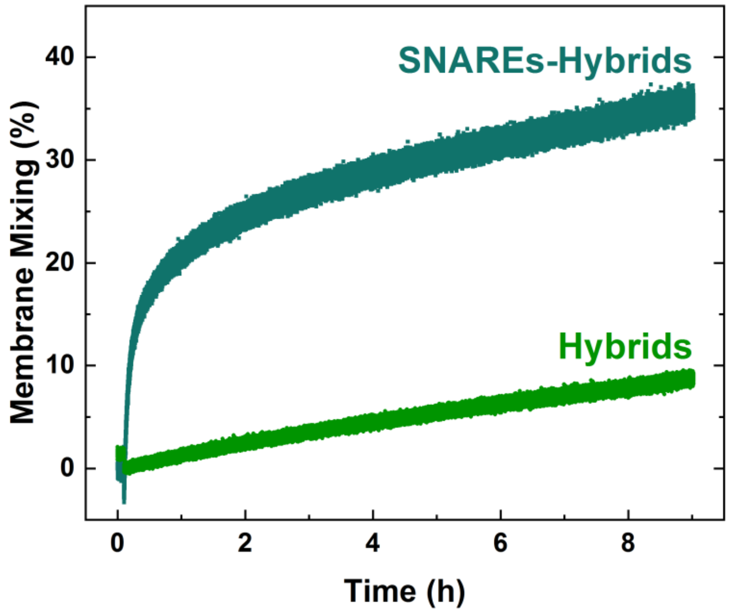

Fig. S7. SNARE-mediated membrane mixing in hybrid vesicles over the course of several hours. In comparison with proteoliposomes, the plateau in SNARE-induced membrane mixing in hybrids was reached after considerably longer period of time (approx. 11.5 h). Proportionally higher total membrane mixing was observed in said time period.

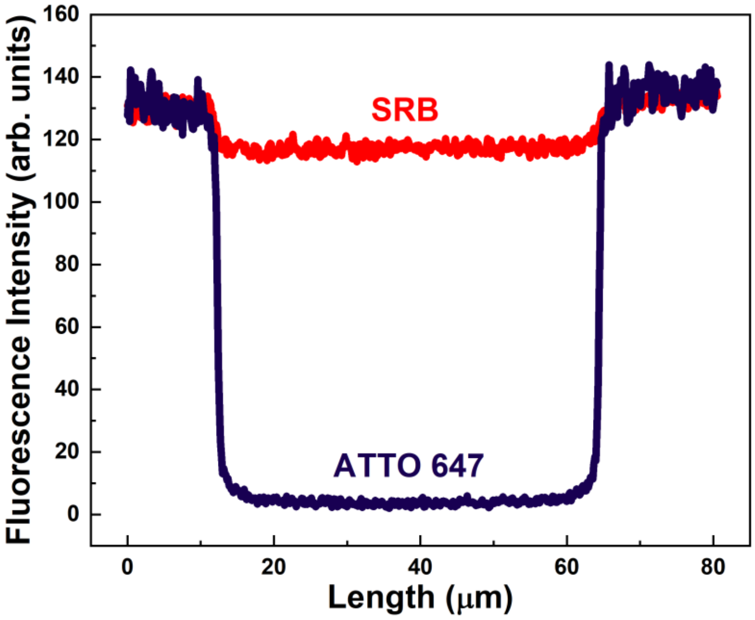

Fig. S8. Dye content of the electroporated polymer vesicle. The polymer GUV, depicted in Fig. 3e was porated in presence of the water-soluble dye sulforhodamine B (SRB), which was able to diffuse into the vesicle through formed pores and was detected in the lumen. Several minutes later, after the closure of micron-sized pores, a second dye (ATTO 647) was added to this vesicle, to check for the presence of submicron pores. ATTO 647N presence was not detected inside the vesicle, indicating complete membrane resealing.

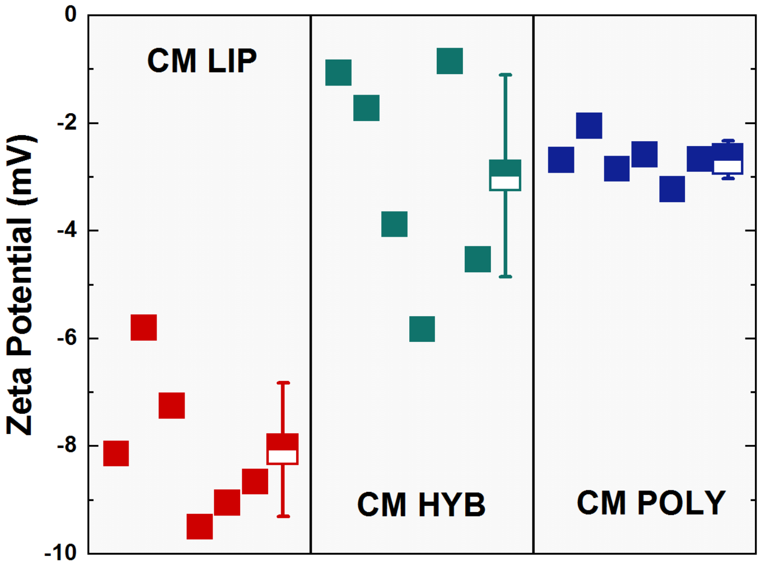

Fig. S9. Zeta potential of vesicles, intended for content mixing in content mixing buffer. Content mixing (CM) lipid (LIP), polymer (POLY) and hybrid (HYB) vesicles were prepared in content mixing buffer containing all cofactors, which were otherwise present in the related measurements (20 mM HEPES [pH 8.0/KOH], 150 mM KCl, 40 mM KH_2PO_4, 73.5 μM ADP, 58.7 nM ATP, 35.2 mM DTT, 0.6 mg ml^{-1} CLSII luciferin/luciferase reagent and 0.2 mM UQ). Zeta potential was measured at 23 °C. Separate measurements with mean values and standard deviations are shown. The composition of vesicles was tailored to match their surface charge in membrane mixing experiments, to enable straight-forward comparison.

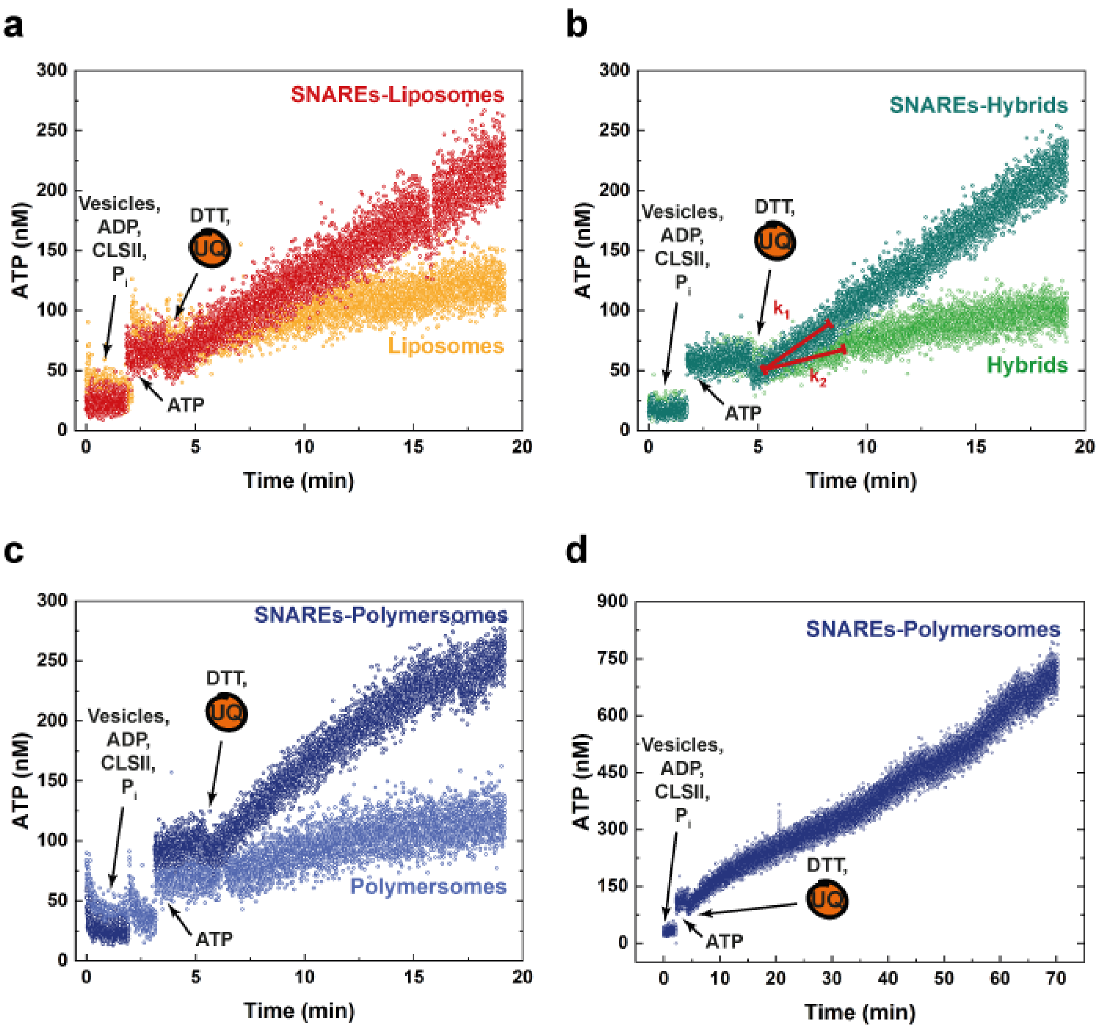

Fig. S10. SNARE-mediated content mixing of lipid, hybrid and polymer vesicles. Comparison between SNARE-induced and SNARE-free content mixing in lipid (a), hybrid (b) and polymer (c) vesicles detected via ATP synthesis. One population of vesicles, containing ΔN complex and ATP synthase, was mixed with a second population, which contained synaptobrevin and *bo*$_3$ quinol oxidase in the presence of cofactors required for ATP synthesis and a luminescence assay (CLSII). After signal calibration with a known amount of ATP, ubiquinone along with the electron donor DTT were added to initiate proton pumping by *bo*$_3$ oxidase. The established proton gradient was used to drive ATP synthesis by the ATP synthase only in the case where both enzymes were integrated in same vesicles (i. e., upon successful fusion). The ATP synthesis rate of SNARE-containing vesicles (k_1) and SNARE-free vesicles (k_2) were determined from the initial rates. While the comparatively lower ATP synthesis in SNARE-free vesicles was seen to subside after several minutes, high ATP synthesis rates were sustained in SNARE-containing vesicles over prolonged period of time. (d) Extended SNARE-induced content mixing in polymer vesicles.

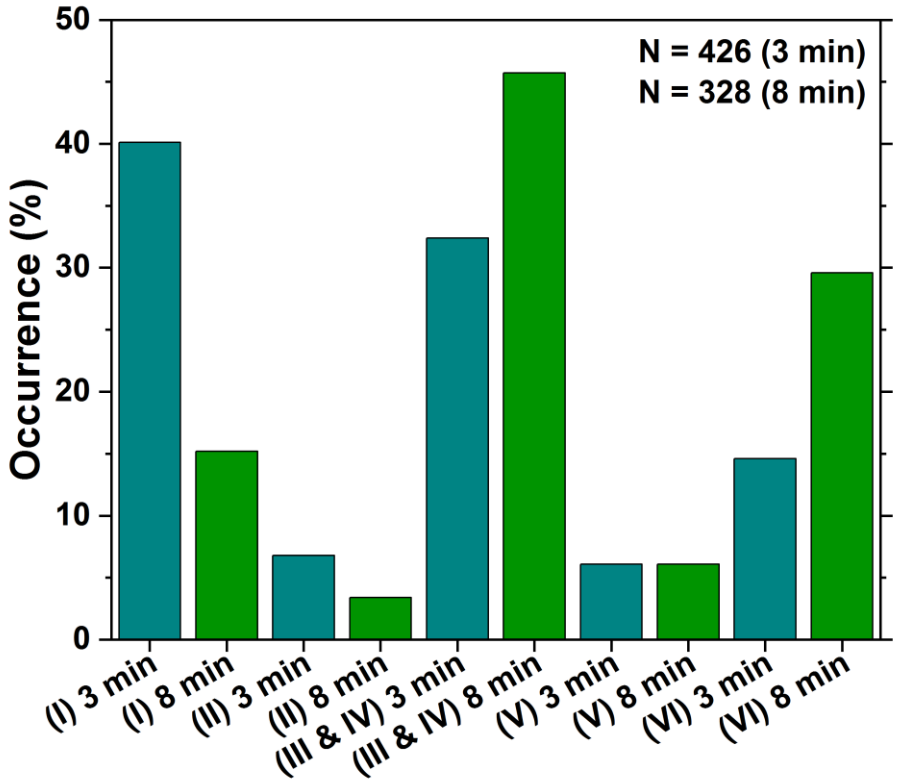

Fig. S11. Intermediates frequency in SNARE-mediated fusion of hybrid vesicles. Shown is the comparison between different intermediates, as described in Fig. 6, observed by cryo-EM after 3 min and 8 min from the initiation of fusion. After 8 min of SNARE-mediated fusion, the early fusion intermediates (I and II) were observed with significantly lower frequency, while the later ones (III-VI) were more frequent. Transient phenomena (hybrid zippering, II, and pore opening, V) were rare occurrences. Denoted with *N* is the number of observed fusion events.

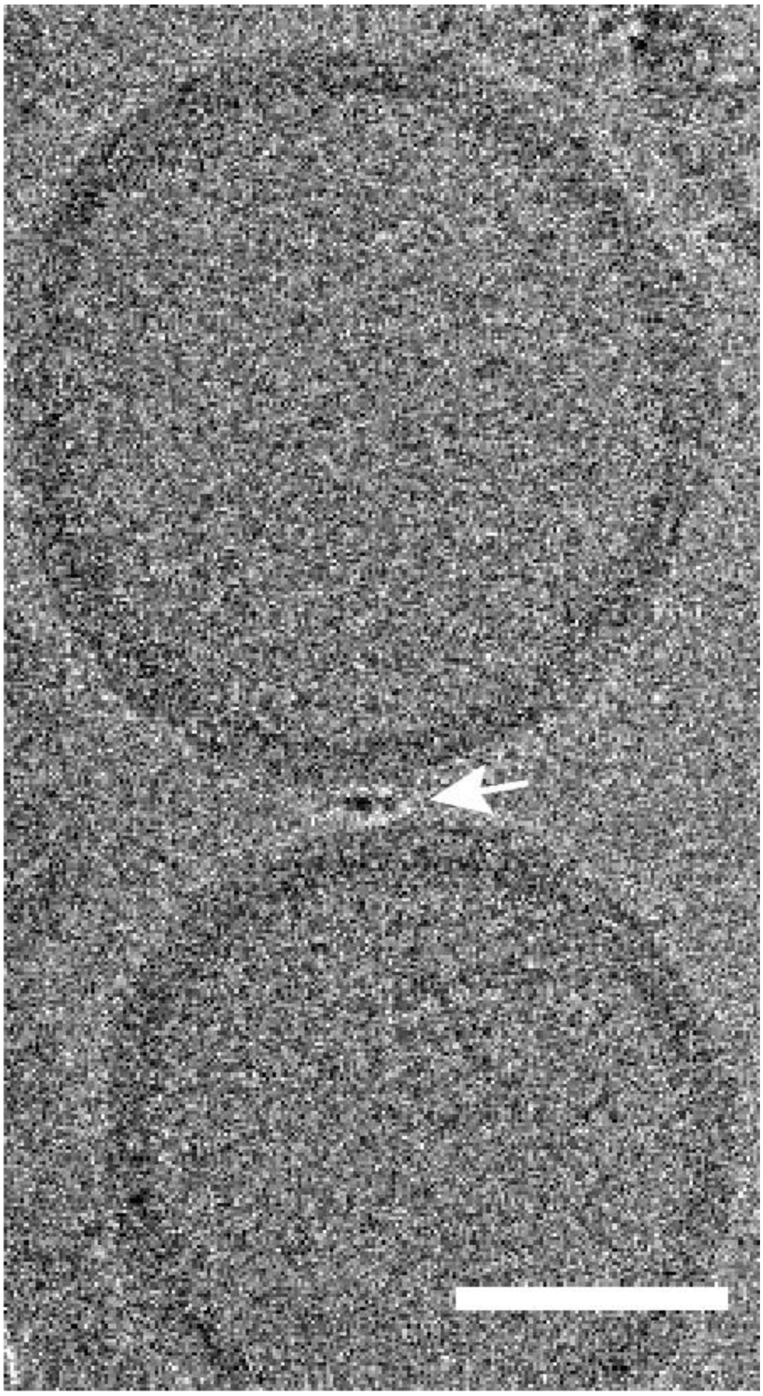

Fig. S12. Docking between SNARE-inserted polymersomes. Vesicles are drawn into close proximity due to the zippering action between synaptobrevin and ΔN complex, reconstituted in separate vesicles. The fully assembled SNAREs fusion complex is indicated with white arrow. Scale bar = 30 nm.

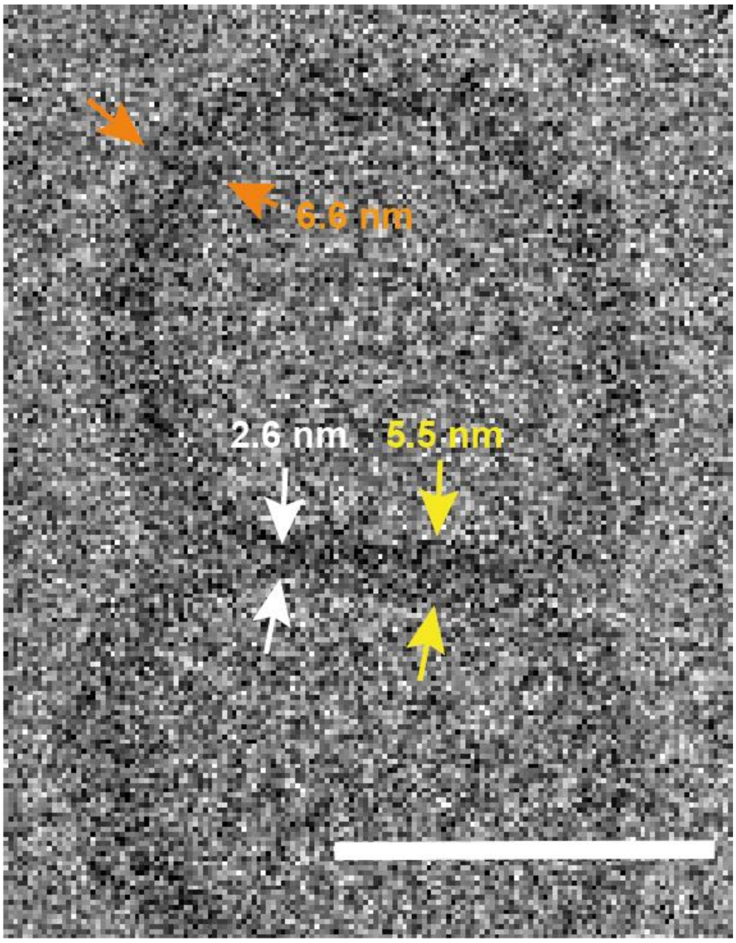

Fig. S13. Diaphragm thinning in fusing polymersomes. Membrane thinning (shown with white arrows) can be observed at the juncture, indicating the plausible location of eventual pore opening. For a comparison, the average diaphragm thickness (yellow) and the average polymer membrane thickness (orange) are also indicated. Scale bar = 30 nm.

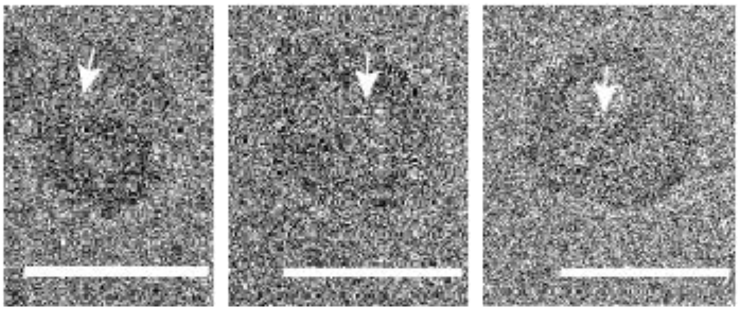

Fig. S14. Unilateral pore opening in polymersomes. Pores are opened at the diaphragm junctures, indicated with white arrows. Scale bar = 30 nm.

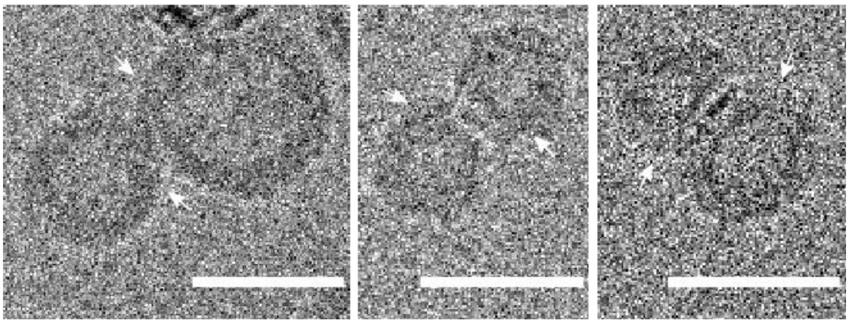

Fig. S15. Hybrid membrane zippering. Point of contact is initially established at one juncture of fusing vesicles and the membrane mixing is propagated diagonally toward the opposite juncture (marked with white arrows). Scale bar = 30 nm.
